# Adoption of an Early Enteral Nutrition Feeding Protocol in Patients Receiving an Allogeneic Stem Cell Transplant

**DOI:** 10.3390/nu18091457

**Published:** 2026-05-01

**Authors:** Nikki Spurgeon, Jana Ponce, Peyton Hainline, Michael Haddadin, Vijaya Raj Bhatt, Christopher Wichman, Emily Thompson, Md Saif Uddin Rashed, Jacque Schwartz, Corri Hanson, Mariah Jackson

**Affiliations:** 1 Department of Clinical Nutrition, Nebraska Medicine, Omaha, NE 68198, USA; pehainline@nebraskamed.com (P.H.); jaschwartz@nebraskamed.com (J.S.); 2 Department of Medical Sciences, College of Allied Health Professions, University of Nebraska Medical Center, Omaha, NE 68198, USA; ckhanson@unmc.edu (C.H.); mariah.jackson@unmc.edu (M.J.); 3 Department of Internal Medicine, Division of Hematology, Fred & Pamela Buffett Cancer Center, University of Nebraska Medical Center, Omaha, NE 68198, USA; mihaddadin@unmc.edu (M.H.); vijaya.bhatt@unmc.edu (V.R.B.); 4 College of Public Health, University of Nebraska Medical Center, Omaha, NE 68198,USA; christopher.wichman@unmc.edu (C.W.); mrashed@unmc.edu (M.S.U.R.); 5 Department of Nutrition and Food Service, Sanford Children’s Hospital, Sioux Falls, SD 57117, USA; emily.thompson4@sanfordhealth.org

**Keywords:** nutrition, enteral nutrition, adult, transplant, graft vs. host disease

## Abstract

Background: Acute Graft versus Host Disease (aGvHD) is a serious complication of allogeneic stem cell transplantation (Allo-SCT) associated with substantial morbidity and mortality. Enteral nutrition (EN) has been associated with improved transplant outcomes, yet standardized early EN practices remain inconsistently adopted across centers. Methods: This retrospective cohort study evaluated the adoption and clinical outcomes of a standardized Day +1 EN protocol in patients undergoing Allo-SCT. The protocol included feeding tube (FT) placement on Day +1 with EN initiated at 25 mL/h. Demographic and clinical data were extracted from electronic health records for patients treated after protocol adoption (post-protocol) and retrospective controls from one year prior (pre-protocol group). Outcomes included successful Day +1 EN initiation, gastrointestinal (GI) complications, FT removal reason, and occurrence and severity of lower GI and overall aGvHD by Day +100 (Modified Glucksberg Criteria). Group comparisons used Welch’s *t*-test and Fisher’s Exact test (*p* < 0.05). Results: The final cohort included 108 patients (67 pre-protocol and 41 post-protocol). Successful Day +1 EN initiation occurred in 95.1% (n = 39) of patients post-protocol versus 4.5% (n = 3) pre-protocol (*p* < 0.001). GI complications and FT removal reason did not differ significantly between groups, and no FTs were removed due to adverse events. The occurrence of lower GI aGvHD was significantly lower post-protocol (12.2% vs. 28.4%, *p* = 0.05). Conclusions: Adoption of a standardized Day +1 EN protocol in Allo-SCT patients was successfully implemented and well-tolerated without adverse FT-related events. The significant difference in lower GI GvHD occurrence in the post-protocol group warrants confirmation of Day+1 EN in patients receiving an Allo-SCT in a future randomized trial.

## 1. Introduction

Acute Graft versus Host Disease (aGvHD), occurring within the first 100 days following allogenic stem cell transplants (Allo-SCT), remains a major source of morbidity and mortality [1]. While aGvHD can impact multiple organ systems, gastrointestinal (GI) involvement is particularly detrimental, associated with longer hospital stays and readmissions, increased mortality, and financial burden [2]. For example, the mortality rate of patients with lower GI aGVHD was 25.7% compared to 7.8% in those without aGvHD (*p* = 0.001) and the cost was significantly higher in those with lower GI involvement than without aGVHD ($256,375 versus $165,622; *p* = 0.014). GI complications of aGvHD include anorexia, early satiety, nausea, vomiting, and diarrhea, which may result in weight loss or malnutrition and ultimately jeopardize transplant success and quality of life [3].

In light of these GI complications, nutrition support is often used to maintain adequate nutritional status. While enteral nutrition (EN) is typically recommended over parenteral nutrition (PN) in the absence of severe GI complications, there has been a lack of standardization across practices lending to a historical preference for PN use [4], as well as caregiver perceptions of PN being less invasive than EN [4,5]. Yet more recently, adequate nutritional intake through EN has been shown to improve outcomes in this population. In a large retrospective cohort study, Beckerson et al. [6] found that patients who received adequate EN had lower non-relapse mortality, improved 5-year survival, and better GvHD-free/relapse-free survival compared to those receiving PN or inadequate intake. Findings such as these have started to reframe EN as not only a critical component of maintaining nutritional status, but perhaps a vital part of aGvHD prevention and management [7]. In fact, a prospective cohort study demonstrated that EN may be protective against aGvHD [8].

Despite the growing evidence of EN being associated with improved patient outcomes [6,9,10], EN remains inconsistently applied across transplant centers. Andersen et al. [11] previously demonstrated the feasibility and tolerability of an early EN protocol in select patients undergoing Allo-SCT, providing important support for its use as a first-line nutritional therapy [12]. Building on this foundation, a critical gap persists in the universal adoption of Day +1 EN as a standard care for all admitted Allo-SCT patients, regardless of conditioning regimen or baseline nutritional status. To address this gap, our study evaluated the adoption of a Day +1 EN protocol in all patients undergoing Allo-SCT and its clinical outcomes.

## 2. Materials and Methods

### 2.1. Study Design and Participants

This retrospective cohort study assessed the feasibility and clinical outcomes of implementing a Day +1 EN protocol in patients undergoing Allo-SCT. The study was conducted in accordance with the Declaration of Helsinki and approved by the Institutional Review Board (or Ethics Committee) of the University of Nebraska Medical Center (#0314-24-EX, 8 September 2024). Electronic medical records were reviewed by trained study personnel using a standardized data abstraction process to collect data for patients who underwent Allo-SCT at a large, academic medical center, between 1 May 2022 and 30 April 2024. Informed consent was waived due to the retrospective nature of the study and the use of data collected under standard hospital admission consent. Adult patients (≥19 years) were eligible if they had a diagnosis of hematologic cancer and underwent Allo-SCT. Exclusion criteria included the following prior to Day +100: disease relapse, stem boost, or death. Patients who expired prior to Day +100 were excluded, as survival beyond this time point was required to assess the clinical outcomes of lower GI and overall aGvHD. Analyses were conducted using an intent-to-treat approach.

### 2.2. Day +1 EN Protocol

**Figure 1:**
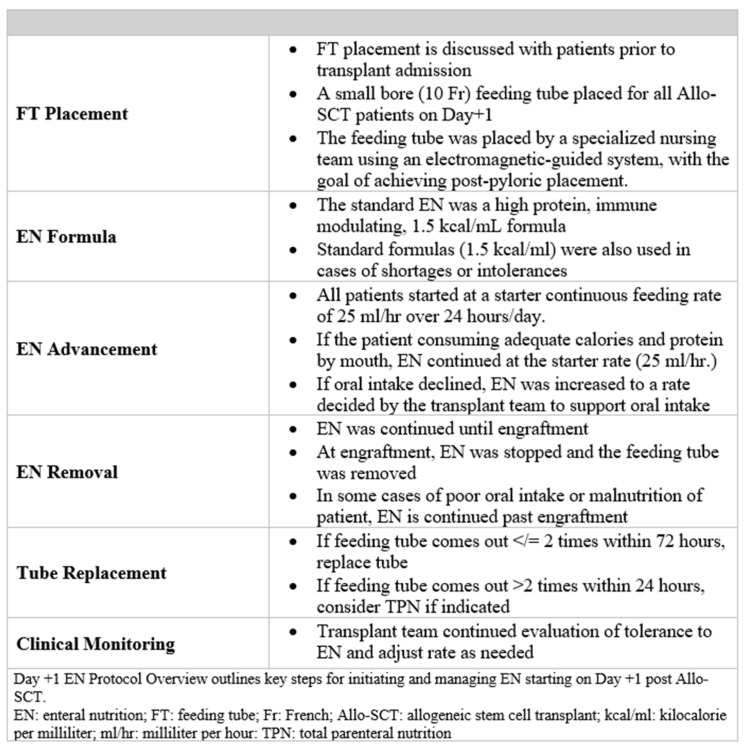
Day +1 EN Protocol Overview.

Based on a review of published literature and in-depth transdisciplinary discussions, the transplant team implemented an early enteral nutrition (EN) strategy beginning in June 2023. The primary exposure in this study was the adoption of the Day +1 EN protocol, which was introduced to standardize tube placement and EN initiation for all patients undergoing allogeneic stem cell transplantation (Allo-SCT) at a large, Midwest Academic Medical Center. This protocol was subsequently incorporated as standard care for all Allo-SCT patients (Figure 1). As part of the Day +1 EN protocol, all patients undergoing Allo-SCT had a small-bore nasogastric feeding tube placed on Day +1. Tube placement was discussed with patients prior to hospital admission. EN was initiated with a high-protein formula providing 1.5 kcal/mL and 94 g of protein/L; however, standard formulas (1.5 kcal/mL and 68 g of protein/L) were used in cases of shortages or formula intolerance. EN was started at a continuous low rate (25 mL/h over 24 h/day) for all patients, as in previous studies [12].

If a patient demonstrated adequate oral intake, EN was maintained at the standard rate (25 mL/h). In cases of declining oral intake, EN was advanced to a goal rate as determined by the registered dietitian nutritionist (RDN). EN was continued through the time of engraftment, at which point it was discontinued and the feeding tube removed. For patients with poor oral intake or malnutrition, EN could be continued beyond engraftment. If the feeding tube became dislodged once or twice within 72 h, it was replaced; however, if dislodgement occurred more than twice within a 24 h period, transition to PN was considered when clinically indicated. RDs monitored patients regularly for gastrointestinal tolerance (e.g., nausea, vomiting, abdominal distension and diarrhea), reviewed enteral nutrition infusion relative to prescribed goals, and adjusted feeding rates as needed in collaboration with the clinical team.

### 2.3. Retrospective Control Group

Patients who received an Allo-SCT from May 2022 to June 2023, one year prior to implementation of the Day +1 EN protocol, served as the retrospective, “pre-protocol” control group for whom clinical data and outcomes were retrospectively abstracted from the EMR using the same definitions as the post-protocol cohort. During this period, EN was initiated on a case-by-case basis at the discretion of the clinical care team.

### 2.4. Protocol and Clinical Outcomes

Outcomes of the Day +1 EN protocol included Day +1 EN, as defined by the successful initiation of enteral nutrition support Day +1 following Allo-SCT, total EN days, advancement of EN past the starter rate of 25 mL/h (yes/no), and FT removal reason (planned, per patient request, patient removed, accidental, or removed due to adverse event).

Clinical outcomes include Allo-SCT hospital admission length of stay (LOS), weight change during the index hospitalization (as calculated by discharge weight minus admission weight), change in BMI from Allo-SCT admission to Day +100, GI complications (no GI symptoms, constipation, diarrhea, vomiting, or more than one symptom), and diagnosis and severity of lower GI and overall aGvHD prior to Day +100 post-transplant.

Diagnosis and severity of lower GI and overall aGvHD were determined from transplant physician documentation in the electronic medical record (EMR). Lower GI aGvHD was defined by physician-documented diarrhea and/or abdominal pain and staged using the Modified Glucksberg Criteria [13], which assign GI stage based on diarrhea volume and symptom severity. The minimum threshold to diagnose Stage 1 lower GI aGVHD includes physician-documented diarrhea with a stool volume ≥500 mL per day, in the absence of an alternative etiology. Overall aGvHD grade was determined by integrating the highest stage across involved organs in accordance with the Modified Glucksberg system. All data were independently reviewed for accuracy by a board-certified transplant physician in hematology and oncology, who confirmed classifications based on the Modified Glucksberg Criteria. Data were collected from transplant day through Day +100, and the highest grade or stage recorded during this period was used for analysis. Diagnosis (yes/no) was defined by any documented occurrence of aGvHD prior to Day +100, while severity was categorized as none (Grade/Stage 0), Grade/Stage 1-2 or Grade/Stage 3-4.

### 2.5. Other Variables

Clinical and demographic variables collected included age, height, admission weight, body mass index (BMI), presence of malnutrition, discharge weight, weight at Day +100, whether enteral nutrition (EN) was advanced beyond a trophic rate, and reason for FT removal (planned removal, removed per patient request, patient removed, accidental, or removed due to adverse event). Hematologic cancer diagnosis was categorized as acute myeloid leukemia (AML), myelodysplastic syndrome (MDS), lymphoma, or other. Transplant-related variables included immunosuppression regimen, donor type, conditioning regimen, and stem cell source. Immunosuppression regimens were classified as: tacrolimus (FK) plus methotrexate (MTX); FK combined with mycophenolate mofetil (MMF) and post-transplant cyclophosphamide (Cy); FK plus MTX and anti-thymocyte globulin (ATG); or other regimens, including sirolimus, corticosteroids, or alternative combinations. Donor type was categorized as matched sibling, matched unrelated, haploidentical, or mismatched unrelated. Conditioning regimens included busulfan/fludarabine (Bu/Flu) myeloablative; Bu/Flu with or without low-dose total body irradiation (TBI); reduced intensity; cyclophosphamide/TBI myeloablative; fludarabine/TBI myeloablative; or other. Stem cell source was classified as peripheral blood or bone marrow.

### 2.6. Statistical Approach

Descriptive statistics between the pre-protocol and post-protocol groups were compared using a Welch’s two-sample *t*-test for continuous variables and a Chi Square or Fisher’s Exact test for categorical variable as appropriate. Protocol outcomes including successful initiation of Day +1 EN in pre- vs. post-protocol groups and total EN days were tested using Chi-Square and an independent samples *t*-test, respectively. Mean EN duration for each group was calculated across the entire cohort, inclusive of patients who did not receive EN; therefore, this measure reflects both the proportion of patients receiving EN and the duration of EN use among recipients, rather than EN duration among recipients alone. Clinical outcomes including GI complications along with lower GI and overall aGvHD stage and grade were determined using Fisher’s Exact test, and an independent samples *t*-test was used for LOS. An alpha of ≤0.05 was considered statistically significant.

## 3. Results

**Figure 2:**
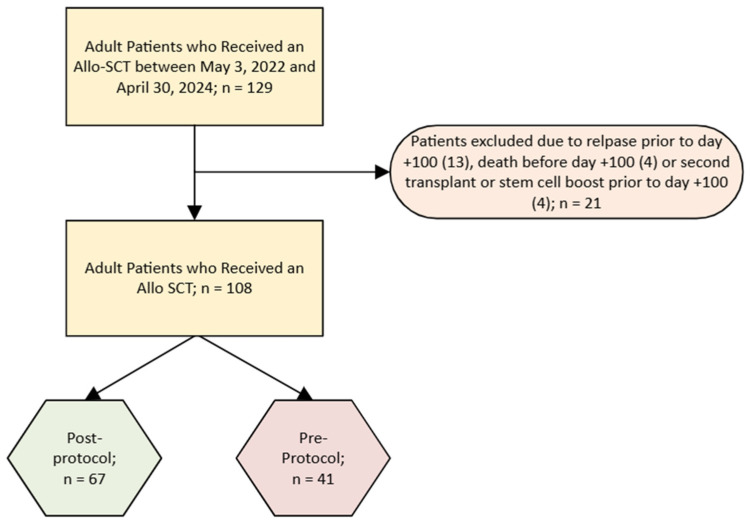
Consort Diagram.

A total of 129 patients underwent Allo-SCT during the enrollment period from May 2022 to April 2024. Twenty-one patients were excluded: 13 due to relapse before Day +100, four due to death prior to Day +100 (three from sepsis and one from relapsed disease who transitioned to hospice care), and four who required a stem cell boost before Day +100 (Figure 2). Our final analytic cohort included 108 patients who received an Allo-SCT, 41 in the post-protocol group and 67 in the pre-protocol group. Overall, the majority of the cohort was male (59.3%), with a mean age of 54.3 (±15.3) years. The predominant hematologic cancer diagnosis was AML (52.8%) with the majority having a matched related donor (65.7%), Bu/Flu with or without low dose TBI, reduced intensity conditioning (50%), and FK+MTX+ATG immunosuppression (59.3%) (Table 1). There were no significant differences in age, sex, BMI, donor type, conditioning regimen, or stem cell source between groups (*p* > 0.05); however, distribution of immune suppression treatment deferred between pre- and post-protocol groups, with FK+MTX used in 10.4% versus 34.1% of patients, FK+MMF+Cy in 13.4% versus 22%, FK+MTX+ATG in 71.6% versus 39%, and other regimens in 4.5% versus 4.9%, respectively (*p* = 0.003).

### 3.1. Protocol-Related Outcomes

Following implementation of the Day +1 EN protocol, initiation of Day +1 EN was successful in 39 patients (95.1%) in the post-protocol group compared with 3 patients (4.5%) in the pre-protocol group (*p* < 0.001) (Table 2). On average, those in the post-protocol group received EN for a mean number of 11.8 (±4.2) days during their Allo-SCT admission while those in the pre-protocol group received EN for 1.43 (±3.92) (*p* < 0.001).

In the post-protocol group, 38 patients (90.5%) had their feeds advanced per RD discretion, while 4 patients (9.5%) remained at the starter rate of 25 mL/h. In contrast, only 8 patients (12.2%) in the pre-protocol group had feeds advanced, 1 patient (1.5%) remained at the starter rate, and 57 patients (86.4%) did not receive any enteral nutrition (*p* < 0.001). FT removal reason did not differ between groups and largely remained in place until planned removal in the pre-protocol (6, 60%) and post-protocol (30, 75%) groups, with no FTs requiring removal due to adverse events.

### 3.2. Clinical Outcomes

The number of patients diagnosed with lower GI aGvHD was significantly lower in the post-protocol group (n = 5; 12.2%) than in the pre-protocol group (n = 19; 28.4%) by day +100 following Allo-SCT (*p* = 0.05) (Table 3). Stage 3–4 and Stage 1–2 lower GI aGVHD occurred in 4 patients (6%) and 14 patients (22.4%) in the pre-protocol group, respectively, compared with 0 patients (0%) and 5 patients (12.2%) in the post-protocol group (*p* = 0.09). Overall, aGvHD was prevalent in both groups with 63 (94%) diagnosed in the pre-protocol and 36 (87.8%) in the post-protocol groups (*p* > 0.05). Although not statistically significant, a higher number of Grade 3–4 and Grade 1–2 cases of overall aGvHD were seen in the pre-protocol group (Grade 3–4: 12 (17.9%); Grade 1–2: 51 (76.1%)) versus the post-protocol group (Grade 3–4: 5 (12.2%); Grade 1–2: 31 (75.6%)) (*p* > 0.05).

No differences in hospital LOS, Allo-SCT admission weight change, or BMI change at day 100 were observed (*p* > 0.05 for all). Notably, there were also no significant differences in GI complications among pre- and post-protocol groups (*p* > 0.05).

## 4. Discussion

This study is the first to introduce a standardized Day +1 EN protocol as the standard of care for all patients receiving an Allo-SCT, regardless of previously considered factors such as conditioning regimen or nutrition status. Despite growing evidence supporting early EN in patients undergoing Allo-SCT, its adoption, particularly starting on Day +1, has been limited, in part due to concerns about patient acceptance and tolerability [7,8,9,12,14]. However, our findings demonstrate that implementing a Day +1 EN protocol was both feasible and well-tolerated. In the post-protocol group, 95.1% of patients successfully initiated enteral nutrition (EN), compared to only 4.5% in the pre-protocol group (*p* < 0.001). Nasogastric tubes were retained until planned removal in 75% of post-protocol patients, with no removals due to adverse events, signaling strong patient tolerability and safety.

Importantly, EN was advanced beyond the starter rate of 25 mL/h in 90.5% of patients in the post-protocol group, indicating that oral intake alone was insufficient to meet estimated calorie and protein requirements and additional EN support was needed as determined by the interdisciplinary team. This underscores the value of early tube placement and timely EN advancement to address the elevated metabolic demands of Allo-SCT. Current guidelines recommend EN initiation when caloric intake remains below 60–70% of estimated requirements for three consecutive days [15]. However, standardized feeding tube placement on Day +1 ensures immediate access to EN when these thresholds are met, whereas prior to protocol implementation, feeding tube placement and EN initiation occurred selectively based on clinical judgment rather than as a routine practice. Early feeding tube placement, such as on Day +1 post-transplant, facilitates prompt intervention and may help prevent malnutrition during hospitalization. In stark contrast, only 12.2% of patients in the pre-protocol group had feeds advanced, highlighting limited EN initiation or progression prior to protocol implementation. These findings suggest that standardized early EN access through a Day +1 protocol provides a greater opportunity to meet nutritional needs by ensuring established access immediately following transplant.

Beyond feasibility, our findings indicate a positive signal for the potential clinical benefit of early EN, with cases of lower GI aGVHD significantly lower in the post-protocol group (12.2%) compared with the pre-protocol group (28.4%; *p* = 0.05). Although differences in severity of lower GI aGVHD were not statistically significant (*p* = 0.09), fewer Stage 3–4 and Stage 1–2 cases were observed post-protocol. These findings are consistent with results published by Seguy et al. [8] and others [12] that have shown a potential protective effect of EN against aGvHD development. Myeloablative conditioning has been shown to disrupt gut barrier integrity in both in vitro [16] and in human models [17], and immunosuppressive agents can cause extensive DNA damage and apoptosis in the GI tract and reduced proliferation of intestinal cells [18]. The resulting increased intestinal permeability and subsequent bacterial translocation have the potential to culminate in systemic infection and reduced survival post-Allo-SCT [19]. Yet, proposed mechanisms of EN include preserving gut integrity by maintaining the mucosal barrier [20], providing continuous stimulation of the gastrointestinal tract to support motility and nutrient absorption, and promoting microbiome diversity and short-chain fatty acid production, which in turn reduce permeability and strengthen tight junction function [21]. Additionally, EN provides an anti-inflammatory nutrient profile that modulates cytokine responses, potentially decreasing intestinal inflammation and mitigating GVHD severity, while also supporting pathways that may limit cellular senescence in the gut epithelium. Together, this demonstrates biologic plausibility and reinforcement of the potential benefit of the protocol observed in our cohort. Although mechanistic exploration is beyond the scope of this paper, these pathways may collectively reduce the risk and severity of lower GI aGvHD in Allo-SCT patients.

Strengths and Limitations: A key strength of this study is the adoption and evaluation of a standardized Day +1 EN protocol in an Allo-SCT population, demonstrating high feasibility, patient acceptance, and tolerability. The protocol was clearly defined and consistently applied, and the findings of this study address a gap in prior research where initiation timing was often variable. We also acknowledge several limitations of this study, including its small sample size, and retrospective single-center design. In addition, data were not collected on the number of feeding tube reinsertion attempts, which limits the assessment of tube-related complications and patient burden. We also did not collect data on parenteral nutrition use, precluding the evaluation of potential differences in supplemental or alternative nutrition strategies between groups. Standards of care may have evolved during the study period, which could have influenced outcomes. Notably, immunosuppressive therapy differed significantly between groups, representing an important source of variability that was not adjusted for in regression models due to the imbalance of lower GI GvHD cases and limited sample size. While the standards of care, which contribute to overall GvHD prevention, continue to evolve, adoption and continuation of a Day +1 EN protocol is warranted given the potential of EN to mitigate the medication-induced GI impact and infection associated with increased bacterial translocation.

While the findings signal improved outcomes for patients following adoption of a Day +1 EN protocol, causality cannot be inferred. Larger prospective studies are needed to assess benefit, and future randomized trials should confirm these observations and explore the mechanistic impact of early EN on transplant-related complications.

## 5. Conclusions

Enacting a Day +1 EN feeding protocol was successfully implemented and well tolerated in patients undergoing Allo-SCT, without an increase in GI complications or adverse FT-related events. These findings signal a potential protective role of early EN in mitigating lower GI GvHD. Given the small sample size of people who developed GvHD, the results are promising, and further investigation including randomized controlled trials is warranted.

## Figures and Tables

**Table 1:** Baseline characteristics among pre-protocol and post-protocol groups.

		Pre-Protocol (n = 67)	Post-Protocol (n = 41)	*p*-Value^a,b^
Age				
	At Admission	55.3 (15.9)	52.7 (14.4)	0.4
BMI				
	At Admission	29.6 (5.4)	29.9 (8.6)	0.8
	Day +100	27.7 (5.4)	27.5 (7.9)	0.9
Length of Stay				
		24.3 (4.5)	24.4 (6.6)	0.9
Sex				
	Male	43 (64.2%)	21 (51.2%)	0.23
	Female	24 (35.8%)	20 (48.8%)	
Diagnosis				
	AML	30 (44.8%)	27 (65.9%)	0.12
	MDS	11 (16.4%)	2 (4.9%)	
	Lymphoma	18 (26.9%)	7 (17.1%)	
	Other	8 (11.9%)	5 (12.2%)	
Malnutrition				
	Malnutrition	7 (10.4%)	6 (14.6%)	0.55
	No Malnutrition	60 (89.6%)	35 (85.4%)	
Immune Suppression				
	FK+MTX	7 (10.4%)	14 (34.1%)	0.003
	FK+MMF+Cy	9 (13.4%)	9 (22%)	
	FK+MTX+ATG	48 (71.6%)	16 (39%)	
	Other	3 (4.5%)	2 (4.9%)	
Donor Type				
	Haplo	7 (10.4%)	3 (7.3%)	0.7
	Matched Sibling	13 (19.4%)	9 (22%)	
	Matched Unrelated	45 (67.2%)	26 (63.4%)	
	Mismatched Unrelated	2 (3%)	3 (7.3%)	
Conditioning Regimen				
	Busulfan Fludarabine ww/o low TBI	37 (55.2%)	17 (41.5%)	0.12
	Myeloablative	18 (26.9%)	19 (46.3%)	
	Other	12 (17.9%)	5 (12.2%)	
Stem Cell Source				
	Bone Marrow	2 (3%)	1 (2.4%)	1.0
	Peripheral Blood	65 (97%)	40 (97.6%)	

^a^ Statistics presented: mean (standard deviation) and count (percent). ^b^ Statistical tests performed: Welch’s two-sample *t*-test and Fisher’s Exact test. FK: tacrolimus; MTX: methotrexate; MMF: mycophenolate mofetil; Cy: post-transplant cyclophosphamide; Other: other regimens, including sirolimus, corticosteroids, or alternative combinations; and nasogastric tube.

**Table 2:** Protocol outcomes among pre-protocol and post-protocol groups.

		Pre-Protocol (n = 67)	Post-Protocol (n = 41)	*p*-Value^a^
Day +1 EN				<0.001
	Yes	3 (4.5%)	39 (95.1%)	
	No	64 (95.5%)	2 (4.9%)	
Days on EN Support				<0.001
		1.43 (4.0)	11.8 (4.2)	
EN Feeding Rate Advanced				<0.001
	Yes	8 (12.2%)	38 (90.5%)	
	No, feeds remained at 25 mL/h	1 (1.5%)	4 (9.5%)	
	Did not receive EN	57 (86.4%)	0 (0%)	
FT Removal Reason				0.5
	Planned Removal	6 (60%)	30 (75%)	
	Per Patient Request	1 (10%)	3 (7.5%)	
	Patient Removed	0 (%)	1 (2.5%)	
	Accidental	3 (30%)	6 (15%)	
	Removed due to adverse event	0 (0%)	0 (0%)	

^a^ Statistical tests performed: Welch’s two-sample *t*-test and Fisher’s Exact test. EN: Enteral Nutrition; FT: Feeding Tube.

**Table 3:** Clinical outcomes among pre-and post-protocol groups.

		Pre-Protocol (n = 67)	Post-Protocol (n = 41)	*p*-Value^a^
Length of Hospital Stay				0.9
	Days	24.3 (4.5)	24.4 (6.6)	
Allo-SCT Admission Weight Change				0.4
	Kg	3.3 (3.5)	4.9 (12.6)	
BMI Change at day 100 (kg/m^2^)				0.3
	At day 100	1.9 (2.2)	2.4 (2.3)	
GI Complications				0.8
	No GI Symptoms	7 (6.5%)	5 (4.6%)	
	Diarrhea	23 (21.3%)	14 (13%)	
	Vomiting	3 (2.8%)	3 (2.8%)	
	Constipation	3 (2.8%)	0 (0%)	
	More than one GI symptom	31 (28.7%)	19 (17.6%)	
Lower GI aGvHD Diagnosis				0.05
	Yes	19 (28.4%)	5 (12.2%)	
	No	48 (71.6%)	36 (87.8%)	
Lower GI aGvHD Severity				0.09
	None (Stage 0)	48 (71.6%)	36 (87.8%)	
	Stage 1–2	15 (22.4%)	5 (12.2%)	
	Stage 3–4	4 (6%)	0 (0%)	
Overall aGvHD Diagnosis				0.3
	Yes	63 (94.0%)	36 (87.8%)	
	No	4 (6.0%)	5 (12.2%)	
Overall aGvHD Severity				0.5
	None (Grade 0)	4 (6%)	5 (12.2%)	
	Grade 1–2	51 (76.1%)	31 (75.6%)	
	Grade 3–4	12 (17.9%)	5 (12.2%)	

^a^ Statistical tests performed: Welch’s two-sample *t*-test and Fisher’s Exact test. Allo: Allogeneic; SCT: Stem Cell Transplant; Kg: kilograms; BMI: Body Mass Index; GI: gastrointestinal; aGvHD: Acute Graft vs. Host Disease.

## Data Availability

The data presented in this study are not publicly available due to privacy and ethical restrictions. The data contain sensitive patient information derived from electronic medical records and are protected under institutional review board (IRB) approval and applicable privacy regulations.

## Abbreviations

The following abbreviations are used in this manuscript:

aGvHD	Acute Graft versus Host Disease
Allo-SCT	Allogeneic stem cell transplant
GI	Gastrointestinal
EN	Enteral nutrition
PN	Parenteral nutrition
